# Mersilene Mesh Brow Suspension: A New Modified Fox's Procedure – Five Years Clinical Experience

**DOI:** 10.4103/0974-9233.51986

**Published:** 2008

**Authors:** Amr Hafez, Mohamed S. Mahmoud

**Affiliations:** From the Research Institute of Ophthalmology, Giza, Egypt

**Keywords:** severe blepharoptosis, ptosis surgery, brow suspension, Mersilene mesh

## Abstract

**Purpose:**

The aiming was to study the long-term clinical outcome and the merit of the author's modification of the fox's procedure.

**Methods:**

Mersilene mesh brow suspension (MMBS) procedure was performed in 50 upper lids with severe blepharoptosis and poor levator function.

**Results:**

The improvement in lid height was evaluated by preoperative and postoperative vertical palpebral aperture measurements and ranged from 2 to 6 mm (average 4 mm). The functional and cosmetically accepted results were maintained in 94% of the lids during mean follow-up of 39.4 months.

**Conclusion:**

In the present non-comparative study we believe that late Mersilene knot extrusion and forehead granuloma formation can be prevented by the modification adopted by the authors.

History of ptosis repair using different techniques and materials dates back to 2000 years.^1^–^9^ Brow suspension (frontalis sling) procedure is universally accepted as the most effective procedure for management of severe blepharoptosis with poor or absent levator function. A major contribution heralding the modern techniques of frontalis suspension was advocated by Payr^1^ who introduced the use of a single central sling of fascia lata. A wide variety of materials have been employed for brow suspension blepharoptosis surgery when an alternative to autogenous fascia lata (AFL) is indicated. Biological and synthetic groups of materials have been tried and all have their proponents.

Synthetic non-absorbable Mersilene macromesh (MM) was first introduced in blepharoptosis surgery by Downes and Collin.^2^ With increasing popularity and usage, extrusion of the knot and forehead granuloma formation have been reported after modified fox pentagon technique.^3^–^6^

The present non-comparative study is focused on the surgical merit of the authors' modification of modified fox pentagon technique in prevention of late knot extrusion and forehead granuloma formation after Mersilene mesh brow suspension (MMBS) procedure over the study period.

## Materials and Methods

Fifty ptotic upper eyelids of 30 patients that had severe ptosis with poor or absent levator function (<4 mm) constituted the subjects of the present study. Mean age was 8.9 years (range 1.5–28 years) and median age was seven years. Ptosis was bilateral in 20 patients including a case of blepharophimosis-ptosis syndrome. It was unilateral in the remaining 10 patients. The etiology was congenital in 49 patients and with third nerve palsy in one patient. Ptosis was recurrent after previous surgery without the use of suspensory material in three patients, two of whom were unilateral.

Pre-operative complete ophthalmological examination including full-face photography was performed in all patients. All cases had normal blinking reflex, corneal sensation and good or acceptable Bell's phenomenon.

**Figure 1:**
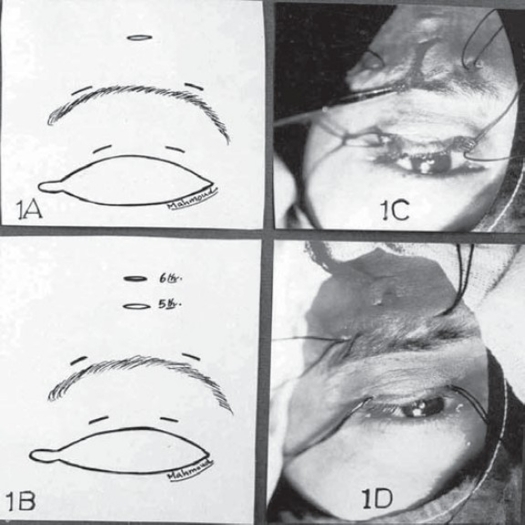
**(A):** Diagram illustrating position of the five horizontal stab incisions in modified Fox's pentagon technique; **(B):** Diagram illustrating the authors' modification of modified Fox's technique. 6th stab is a central brow incision 5 mm above and parallel to the original 5th central superior brow incision; **(C):** Mersilene strands coming through the original 5th superior brow incision (The original modified Fox's pentagon technique). Notice: 4 pieces of black silk thread is temporarily inserted in stab incisions. **(D):** The final position of the sling with Mersilene strands rethreaded through the 5th incision to appear through the 6th central brow incision (Authors' modification).

The surgical procedure of MMBS was the authors' modification of the original modified fox pentagon technique described by Downes and Collin.^2^ Moreover four pieces of 4/0 black silk thread were temporarily inserted under the strips of MM in the lid and brow incisions and appearing freely outside the incisions as adopted by Hintschich et al.^3^ Manipulation of the inserted black silk thread aids adjusting the lid contour and height as shown in Figure 1A, B, and C.

The authors' modifications adopted in the present study include two main points. First, addition of 6th deep brow incision which is taken as horizontal stab that goes down to the periosteum, parallel to and 5 mm above the original 5th deep superior brow incision described by Downes and Collin^2^ as shown in Figure (1-B & C). Second, the sling is cut from a sheet of MM. (supplied by Ethicon) in measurements (3 - 4 × 150 mm) instead of (7 × 150 mm) as originally published by Downes and Collin.^2^

On the completion of procedure, the desired lid height and contour was obtained after pulling on both ends of the sling 4/0 black silk threads were pulled out and the two strips of the mesh were held taut side by side and secured using 5/0 Ethibond sutures transfixing the mesh just within the 5th brow incision. No attempt to secure the terminal ends of the mesh by a knot formation was made. The terminal ends of the mesh were then rethreaded through the original 5th superior brow incision to appear in the uppermost 6th incision (Figure 1–D). Care was taken to direct the mesh-threaded needle first perpendicular till the periosteum then vertically just over the periosteum of the frontal bone so that the mesh tissue was deep to frontalis. The excess mesh was trimmed, while pulling and the sling was allowed to retract into the deep tissue of the forehead. The four brow incisions were closed in single layer with interrupted 6/0 vicryl. A traction suture was placed in the lower lid and dressing with topical antibiotics was applied for three days.

Postoperative systemic antibiotics were administered for one week. Topical antibiotics were prescribed for one week. Patients were assessed on the second day, at 1, 2, 6 weeks and then 3, 6 months and then yearly

## Results

The MMBS was performed on 50 upper eyelids with severe blepharoptosis and poor or absent levator function. Mean age was 8.9 years (range 1.5 – 28 years) and median age was seven years. Mean follow-up was 39.4 months (range 30 - 54 months) and median follow-up was 37 months.

The cosmetic result in the present study depended on the subjective estimation of the authors and the patients or their parents. Surgical outcome was judged as good, fair or poor, based on the criteria described by Beard,^7^ and was found to be acceptable during the first 6 – 24 weeks. A good result was defined as a postoperative lid level resting 2 to 3 mm below the superior corneal limbus without the use of the frontalis muscle in a bilateral case, or within 1 mm range of the opposite normal lid in a unilateral case. A fair result was defined, as a lid level in the same position as described above with the use of frontalis muscle achieving as cosmetically acceptable position.

A poor result was defined a postoperative lid level 4 mm or more below the superior corneal limbus, even with maximal use of frontalis muscle.

Improvement was evaluated by pre- and post-operative vertical palpebral aperture measurements. Good to fair results were obtained with the upper eyelid levels at 2 to 3 mm below the upper limbus in 47 lids (94%). The remaining three lids (6%) had poor result and were reoperated six months postoperatively (including a case of 3rd nerve palsy) because the cosmetic results were unsatisfactory.

**Figure 2:**
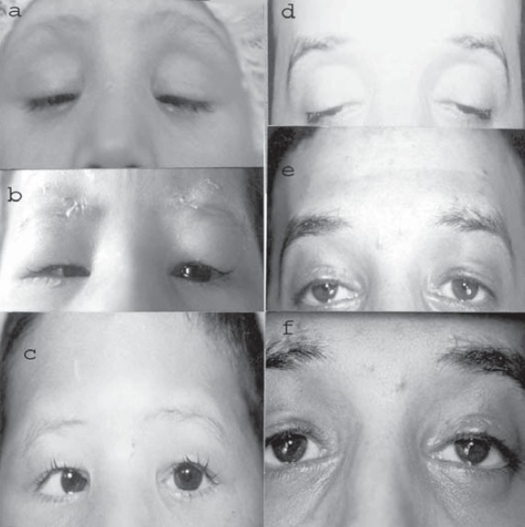
Bilateral MMBS procedure for congenital ptosis. Pre-operative, immediate post-operative with vicryl sutures in place and late post-op (5-yr-old boy: **a, b,** and **c**; 25-yr-old male: **d, e,** and **f**).

**Figure 3:**
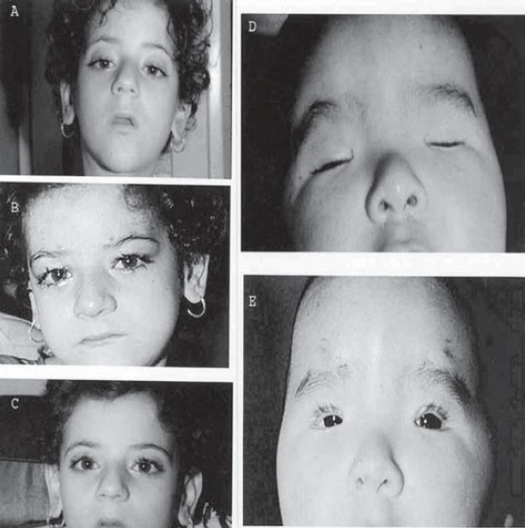
Pre- and post-operative appearance after bilateral MMBS procedure for congenital ptosis. **(A)** Pre-operative with chin-up posture. **(B)** Early postoperative with vicryl sutures in place. **(C)** Late post-operative with chin–up posture already resolved. **(D and E)** Blepharophimosis - ptosis syndrome Pre- and post-operative appearance. Notice fine scar marks at incisions sites.

**Figure (4):**
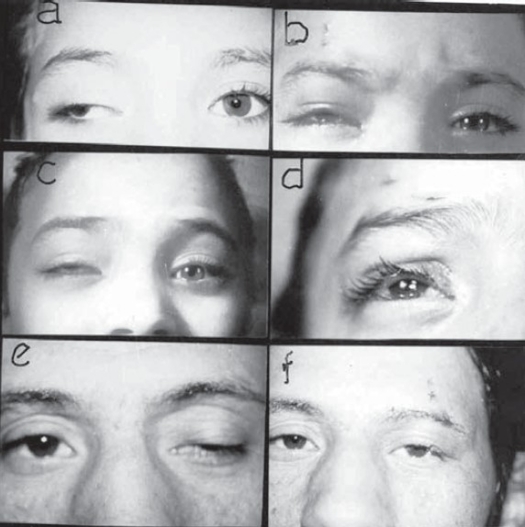
unilateral MMBS procedure.(**a** and **b**): 7-yr-old girl with paralytic ptosis due to 3rd nerve palsy before and after squint and MMBS procedure. **(c)** Cosmetically unacceptable correction 6 months post-op. **(d)** recent re-operation with new MM strip. (**e** and **f**) 20-yr-old male before and after surgery with vicryl sutures in place.

**Figure (5):**
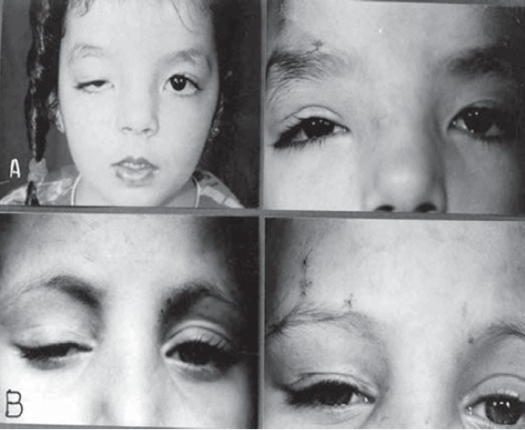
Preoperative and early postoperative appearance after MMBS procedure for unilateral congenital ptosis recurrent after previous ptosis surgery without suspensory material. **(A)** 5-yr-old girl recurrent for 3rd time. **(B)** 8-yr-old boy recurrent for 2nd time.

The functional and cosmetic outcome was satisfactory in bilateral cases (Figures 2 and 3) as well as in unilateral cases (Figures 4 and 5). Post-operative cosmetically acceptable asymmetry on down gaze (lid lag) was observed in only some unilateral cases. All cases had a smooth lid contour, without marginal irregularities or peaking of the skin over the sling. Temporary dimpling and skin wrinkling at stab incision sites disappeared gradually. Three patients had clinically insignificant lagophthalmos during sleep. However, since all cases had a good or acceptable Bell's phenomenon, prophylactic topical lubricant eye ointment guarded against exposure keratopathies.

There were no instances of late Mersilene knot extrusion or forehead granuloma formation, stitch granuloma, fistula, or wound infection in the follow up period of study.

## Discussion

The use of frontalis muscle to elevate the lid is now the treatment of choice for management of severe blepharoptosis with poor or absent levator function however, there is no consensus on the ideal choice of material. Materials that have been advocated in brow suspension ptosis surgery include grafts and synthetics. Fresh autogenous fascia lata (AFL) remains the most popular and the most effective. It achieves a permanent effect, retaining its cellular viability.^10^,^11^ However, it has certain disadvantages as a second operative site anatomically unfamiliar to most ophthalmologists is required to obtain (AFL) and children under the age of three may not have enough tissue.^12^ Other grafts used include stored irradiated fascia, lyophilized fascia,^13^ skin,^14^ orbicularis muscle,^15^ strips of frontalis muscle and corrugator supercilii,^9^ levator tendon,^16^ extensor tendons,^17^ and fresh or preserved sclera^18^ but the results are less than satisfactory.

Some of synthetic sling materials include silicon rods or bands,^19^,^20^ catgut, nylon, silk, or polyester sutures^9^ and poly-filament cable-type suture,^21^ metals such as gold, silver, and platinum,^9^ as well as combined carbon polyester ligaments,^22^ and Gore-tex soft tissue patch.^23^ Most synthetic materials have a significant incidence of slippage as a result of inadequate tissue incorporation. The concept of an open mesh that is integrated into the host tissue is a desired one. Mersilene macromesh applied in the present study is synthetic non-absorbable polyester manufactured by a machine knitting process, which interlocks individual fiber junctions. This feature prevents unraveling and disruption of adjacent fiber junctions when the mesh is cut. It is inexpensive, readily available, easily prepared, handled and implanted. The material is biochemically stable, reautoclavable and durable maintaining its tensile strength with time. MMBS have been attempted as an easily performed procedure in infants less than one year of age.^24^

In the present series, with authors' modification the functional and cosmetic outcome has been found to be quite satisfactory with good to fair result in 94% of the lids. Young children under two years of age had achieved normal or near normal eyelid position by this one-step procedure. All had their chin-up posture resolved with time. Poor result requiring reoperation was reported in three out of the fifty operated lids (6%). None of the cases were complicated by late Mersilene knot extrusion, forehead granuloma formation or wound infection, fistula or mesh extrusion. The desirable lid height was obtained within six weeks post-operatively when the operative edema had resolved and the normal orbicularis-frontalis muscles tone had regained. The lid height was maintained throughout the whole mean follow up period 39.4 months (30 to 54 months) without loss of tensile strength.^25^ In the present study, the lid height adjustment was done intraoperatively to the required level as determined for each patient without the need to elevate the lid to the level of or just above the superior limbus as recommended in several reports.^24^,^26^

The results of the present study compares favorably with the currently published studies using MM.^2^,^27^,^3^,^6^,^24^,^26^,^28^,^29^ For example, Downes and Collin in their first results reported satisfactory lid positions maintained in 22 out of 23 cases during mean follow up of 15 months.^2^,^27^ Hintschich et al^3^ reported good and moderate results with satisfactory lid position in 96% of 76 lids in these two groups with mean follow up of 20 months. Similarly, Can et al^6^ reported success in 22 out of 23 lids during mean follow up of 25 months. Moreover, Lam et al^24^ reported encouraging results in all ten pediatric cases before one year of age during mean follow up of 40.3 months. Furthermore, Gabrieli et al^26^ reported a significant stable improvement in lid height in all operated 20 lids during a mean follow up of 18 months. In comparative study with (AFL), El-Toukhy et al reported no complications in 46 operated lids using MM.^28^ Elder confirmed that no changes in lid position had occurred in all 17 operated lids from the second week until the final assessment.^29^

As expected, in the present study, post-operative lid lag on down gaze was observed in some unilateral cases. It is the result of hindrance in complete release of the operated lid. However, it was so cosmetically acceptable and none of the cases required any simultaneous surgery on the sound fellow eye as recommended by several authors.^30^,^31^ In the present work, poor result requiring re-operation was reported in three lids (6%) 6 months post-operatively. Paralytic ptosis with 3rd nerve palsy was the etiology in one case, while the remaining two were recurrent after previous surgery without suspensory material. We do believe that this early recurrence is due to inadequate lid height adjustment at the time of surgery or due to gradual slippage of the mesh through the palpebral tissue as a result of inadequate tissue incorporation with subsequent loss of the required lid height. All the abovementioned recurrent cases were reoperated with a new modified MM strip (3 - 4 × 150 mm) with final functional and cosmetically acceptable results.

Several authors have reported forehead granuloma formations with or without extrusion of MM knot. For example, Hintschich et al^3^ in their series reported long-term results and described extrusion, granuloma formation and infection in 9 out of 66 lids (13.6%) during mean follow up of 23 months. Mutln et al^4^ had overall rate of 6.3% extrusion or granuloma formation. In their opinion it may be related to either foreign body reaction secondary to MM or superficial placement of the sling material. Unfortunately, higher incidence rate (20%) of soft tissue complications (extrusion, granuloma, and infection) was reported by Mehta et al during mean follow up of 32 months.^5^ Can et al reported herniation of the Ethibond – tied mesh ends out of the superior brow incision site in one out of 23 lids (4.3%) during mean follow up 25 months.^6^ Extrusions of MM used as an upper lid spacer have been also reported in 20% of cases.^32^ On the other hand El–Toukhy in comparative study with AFL reported no complications in 46 eyelids operated upon with MM.^28^ Detailed review of the original abovementioned articles revealed that some authors had secured the two ends of the (7 × 150 mm) MM in complete single knot^3^,^4^ while others applied half knot^5^ a step that was not originally mentioned by Downes and Collin.^2^ In the present study we advocate a narrow MM strip (3 – 4 × 150 mm), and stress that knot formation be avoided and recommend additional 6^th^ superior deep brow incision. This modification creates a musculocutaneous tissue strip between the 5^th^ and 6^th^ central brow incisions which acts as a natural tissue coverage overlying the deeply embedded knot and MM terminals that can prevent late knot extrusion and granuloma formation.

In the present study with the meticulous aseptic technique, proper wound closure and routine use of systemic antibiotics for one week, none of the cases were complicated by wound infection, although the MM was not rinsed in antibiotic solution before implantation as originally stressed by Downes and Collin.^2^

Incorporation of MM into the host tissue with regularly arranged parallel collagen bundles are documented in animal and clinical studies of several authors.^24^,^27^,^33^,^34^

In conclusion we recommend MMBS procedure with modification as an alternative for patients with severe ptosis and poor levator function who are not primarily suitable candidates for AFL. It may be a promising one-step procedure in young children under two years of age. This study suggests that late Mersilene knot extrusion and forehead granuloma formation can be prevented by our modification. However, because it is a synthetic material, the probability of extrusion or granuloma formation as a natural reaction to a biological system should be entertained.
